# EFFECTS OF RESTRICTIONS IN COMMUNITY-BASED CARE SERVICES ON FAMILY CAREGIVERS DURING THE COVID-19 PANDEMIC

**DOI:** 10.1093/geroni/igae098.2330

**Published:** 2024-12-31

**Authors:** Ayumi Honda, Yin Liu, Elizabeth Fauth, Sumihisa Honda

**Affiliations:** St. Mary’s College, Kurume, Fukuoka, Japan; Utah State University, Logan, Utah, United States; Utah State University, Logan, Utah, United States; Nagasaki University, Nagasaki, Nagasaki, Japan

## Abstract

This mixed-methods study aimed to examine the effects of restrictions in the utilization of community-based care services on family caregivers during the COVID-19 pandemic. We recruited from community-based care services 200 family caregivers who were living with an older care recipient. We first conducted thematic analyses based on open-ended responses to questions regarding the impact of restrictions in the utilization of community-based care services. We then extracted themes to examine and determine patterns across caregiving characteristics. Next, we conducted linear regression analysis to examine associations between impact of restrictions and caregiving contexts. Findings from the thematic analyses identified 11 themes regarding the impact of restrictions in the utilization of community-based care services. Most frequently reported impact included “the family caregiver cannot bathe the older person at home” and “the family caregiver cannot go to work/income decreases”. Additionally, findings from regression analysis suggested that caregiver employment (β = 0.292, SE = 0.122, p =.02), increased caregiving tasks (β = 0.134, SE = 0.051, p =.01), and more behavioral and psychological symptoms of dementia (β = 0.088, SE = 0.037, p =.02) were associated with harmful impacts resulting from restrictions in the utilization of community-based care services. To conclude, family caregivers identified common areas where they felt ill equipped to provide home care during the COVID-19 pandemic. The study informs priorities areas of needed external support, resources, or training that family caregivers may need in planning short- or long-term emergency preparedness.

